# Applications of machine learning tools for ultra-sensitive detection of lipoarabinomannan with plasmonic grating biosensors in clinical samples of tuberculosis

**DOI:** 10.1371/journal.pone.0275658

**Published:** 2022-10-25

**Authors:** Yilun Huang, Charles M. Darr, Keshab Gangopadhyay, Shubhra Gangopadhyay, Sangho Bok, Sounak Chakraborty

**Affiliations:** 1 Department of Statistics, University of Missouri, Columbia, Missouri, United States of America; 2 Department of Electrical Engineering & Computer Science, Center for Nano/Micro Systems & Nanotechnology, University of Missouri, Columbia, Missouri, United States of America; 3 Department of Electrical & Computer Engineering, University of Denver, Denver, Colorado, United States of America; Public Library of Science, UNITED KINGDOM

## Abstract

**Background:**

Tuberculosis is one of the top ten causes of death globally and the leading cause of death from a single infectious agent. Eradicating the Tuberculosis epidemic by 2030 is one of the top United Nations Sustainable Development Goals. Early diagnosis is essential to achieving this goal because it improves individual prognosis and reduces transmission rates of asymptomatic infected. We aim to support this goal by developing rapid and sensitive diagnostics using machine learning algorithms to minimize the need for expert intervention.

**Methods and findings:**

A single molecule fluorescence immunosorbent assay was used to detect Tuberculosis biomarker lipoarabinomannan from a set of twenty clinical patient samples and a control set of spiked human urine. Tuberculosis status was separately confirmed by GeneXpert MTB/RIF and cell culture. Two machine learning algorithms, an automatic and a semiautomatic model, were developed and trained by the calibrated lipoarabinomannan titration assay data and then tested against the ground truth patient data. The semiautomatic model differed from the automatic model by an expert review step in the former, which calibrated the lower threshold to determine single molecules from background noise. The semiautomatic model was found to provide 88.89% clinical sensitivity, while the automatic model resulted in 77.78% clinical sensitivity.

**Conclusions:**

The semiautomatic model outperformed the automatic model in clinical sensitivity as a result of the expert intervention applied during calibration and both models vastly outperformed manual expert counting in terms of time-to-detection and completion of analysis. Meanwhile, the clinical sensitivity of the automatic model could be improved significantly with a larger training dataset. In short, semiautomatic, and automatic Gaussian Mixture Models have a place in supporting rapid detection of Tuberculosis in resource-limited settings without sacrificing clinical sensitivity.

## Introduction

Tuberculosis (TB) and many other highly infectious diseases took a back seat in research over the last year as the world shifted focus to respond to the coronavirus (COVID-19) pandemic, but the disease lost none of its importance in infections, mortality, or global impact. In fact, recent reports of co-morbidity of TB with COVID-19 across the globe highlight the need for accurate, sensitive infectious disease testing [1–6]. Subclinical TB infected persons may have sufficient pathogen load to be contagious and yet go undetected for unacceptable periods of time, risking further spread and worsening condition prior to onset of treatment. Moreover, patients undergoing treatment may test negative by one or more methods yet still have a low level active pathogen load, leading to recurrence after cessation of treatment [7, 8]. While testing will continue to improve with increased knowledge about this particular disease, the current epidemic has exposed many weaknesses of both gold standard and emerging diagnostic methods for the detection of TB such as cell culture and reverse transcriptase polymerase chain reaction (RT-PCR) testing that have long existed and warrant greater attention given the current need for increased detection capacity. Addressing this need requires sensitivity of unprecedented scale, since low pathogen loads result in contagious subclinical infection and sampling noninvasive body fluids may have even lower concentrations of critical biomarkers of disease. Ideally, confident positive identification of a single molecule (SM) of a disease biomarker can be achieved.

To that end, single molecule fluorescence imaging (SM imaging) is a powerful optical tool to identify sub-picomolar molecular concentrations and to decouple unique SM behavior from the averaged behavior of bulk fluorescence [9, 10]. SM imaging has been used to study a number of biomolecular systems, including protein folding [11], cellular endocytosis and exocytosis, biomolecular interaction through fluorescence resonance energy transfer (FRET) [12, 13], analysis of local environmental effects, and superresolution imaging [14–17]. SM imaging has so far been limited by the expensive optics required for its application, such as total internal reflection (TIR) or confocal optics. Moreover, SM imaging studies often generate immense physical quantities of data, which trained experts must then painstakingly analyze to extract meaningful information from the fluorescence while filtering out data resulting from noise and outliers such as nonspecific binding, dust, autofluorescence, and background. One nearly ubiquitous feature of SM imaging with organic dyes, quantum dots, and fluorescent polymers is single-molecule blinking or flickering behavior [18]. Blinking is often seen as detrimental as it disturbs calculations of FRET efficiency by removing one or more dyes from the emissive state and negates other fluorescence modulating effects, and so most researchers attempt to reduce or remove blinking behavior through addition of stabilizing reagents. However, blinking can be used as a detection mechanism under appropriate conditions.

One significant drawback of using fluorescence blinking as a detection modality is the associated signal-to-noise ratio. Recently, our group has developed a cost-effective plasmonic grating platform to detect and analyze chemical and biological molecules down to the SM level using only an upright epi-fluorescence microscope [19–23], replacing expensive total internal reflection (TIR) or confocal optics. Plasmonic gratings rely on a property of noble metals in which they convert incident photons into standing electromagnetic (EM) waves at the surface of the metal-dielectric interface in a process known as surface plasmon resonance (SPR) [24–26]. Proper shaping of the metal into a nanoscale grating structure provides additional optical momentum that allows frequencies other than the plasma frequency to be coupled to the surface plasmons. In effect, a grating of ~400 nm spacing (i.e., pitch) allows coupling of visible wavelengths of light at angles close to normal without the use of a high-index prism. The resulting electric field can interact with and excite nearby fluorophores, which subsequently transfer energy nonradiatively to the grating to form a radiative plasmon, which is emitted from the grating at a specific angle and wavelength (surface plasmon coupled emission, SPCE). Through proper tuning, the combined SPR/SPCE effect increases the observed emission intensity by up to 200× the intensity of the same fluorescent molecules on a non-plasmonic substrate [20, 21, 27]. This intrinsic signal amplification greatly enhances the signal-to-noise ratio of incoming light from observed objects, increasing the contrast of collected fluorescence images and, thus, enabling much lower limits of detection, including down to the SM level [21, 28, 29]. One advantage of this enhancement is reduced time-to-diagnosis of highly infectious disease at small local quantities in the clinical specimen taken and from complex clinical specimens that may have interfering analytes for RT-PCR type tests, reducing Type II errors (i.e., false negatives) of subclinical or early clinical infected persons.

Much of any large set of SM imaging data ends up discarded or ignored in favor of molecules that exhibit the featured mechanism of interest (e.g., FRET identifying colocalization of molecules of interest). However, the remaining dataset could still provide meaningful information if trends or distributions of molecular behavior could be identified. Just considering blinking, the intensity of on-states, duty cycle or “on time”, frequency of intensity oscillation, and other effects might be used to classify a molecule and its local environment with meaningful scientific explanation. In principle, machine learning gives computer systems the ability to learn without explicit programming by creating algorithms that can learn from a large data set and make predictions on the data [30]. With the distinct advantage of handling a large amount of data in relatively short periods of time compared to manual inspection, machine learning has been applied to medical image processing and biomedical diagnostics. In particular, machine learning techniques have been used to identify and model patterns in stochastic SM imaging data investigating time series molecular dynamics in response to local environmental effects [31, 32]. Recent work by Wu [33] has yielded promising results regarding hierarchical and density-based clustering in analyzing SM data. Together with the classification approach, our statistical machine learning approach distinguishes SM from background at a level of accuracy similar to manual counting while relieving the workload of a trained human expert. Further, the method developed herein operates at a very high rate of speed compared to manual counting and other available machine learning algorithms, completing the detection task in under a minute whereas manual counting on the same dataset takes several hours.

## Materials and methods

### Grating preparation

Silver plasmonic gratings were prepared by soft lithography process similar to [19–23]. A silicone stamp was prepared by curing 5:1 ratio Sylgard^®^ 184 polydimethylsiloxane (PDMS, Gelest) over a halved, cleaned HDDVD for 24 hours at 50 °C and 55% relative humidity. Meanwhile, plain glass microscope slides (Corning) were cleaned by successive bath sonication in acetone, methanol, and deionized water, and dried under flowing nitrogen. Cleaned slides were then soaked for 10 minutes in 3:1 H_2_SO_4_:H_2_O_2_ (Piranha solution), washed twice in fresh deionized water, rinsed under copious flowing deionized water, and dried under flowing nitrogen. Cured PDMS was cut into 1” square slabs, spin-coated with 3% w/w GR650F polymethylsilsequioxane (PMSSQ, Techneglas) in ethanol, and stamped onto the cleaned glass slide. The gratings were then vapor-treated with 1:1 3-aminopropyltriethoxysilane (APTES) in ethanol, pre-baked at 60 °C for 3 hours, and baked at 400 °C for 1 hour. A 100 nm silver layer was sputtered onto the gratings (AJA RF Magnetron) and coated with 10 nm alumina by low-temperature atomic layer deposition.

### Assay preparation

Assays were prepared in a method similar to [23, 34]. Activation buffer was prepared by adding 11 mg/mL sulfo-N-hydroxysuccinimide (Sulfo-NHS) and 4 mg/mL 1-ethyl-3-(3-dimethylaminopropyl)carbodiimide (EDC) to pH 6.0 2-(N-morpholino)ethanesulfonic acid (MES) in deionized water. Meanwhile, alumina-coated silver gratings were exposed to 30 s 7 W CO_2_ plasma and a ProPlate^®^ 24 well slide adapter (Grace Bio-Labs) was clipped onto the slide. Activation buffer was aliquoted to 75 μL per well and incubated at room temperature for 10 minutes. BPM102 anti-lipoarabinomannan (anti-LAM) antibody (Intellectual Ventures, Bellevue, Washington, USA) was diluted to 20 μg/mL in pH 8.0 MES, aliquoted to 75 μL per well (total volume 150 μL), and incubated at 4 °C overnight. After equilibrating to room temperature, the antibody solution was decanted and the slides washed with 0.1% v/v Tween-20 in pH 7.4 phosphate buffered saline (wash buffer) for 5 minutes. Wash buffer was decanted and the wash repeated in triplicate. After washing, blocking buffer (3% w/v bovine serum albumin in PBS-T) was aliquoted to 150 μL per well, incubated for 1 hour, decanted, and washed in triplicate with wash buffer. Patient urine samples and spiked urine control samples were thawed and aliquoted to 100 μL per well (n = 3 per patient) and incubated at 4 °C for 2 hours. The samples were decanted and the slide washed in triplicate with wash buffer as above. Then, BPM101 biotinylated anti-LAM antibody (5 μg/mL in PBS) was aliquoted to 100 μL per well and incubated at 4 °C for 2 hours. This solution was decanted, the slides washed in triplicate with wash buffer, and replaced with 10 μg/mL AlexaFluor 568-labeled Streptavidin aliquoted to 100 μL per well and incubated at 4 °C for 2 hours. Samples were again washed with wash buffer in triplicate, rinsed with plain buffered saline, and deionized water. The slide modules were removed and replaced with 1 in × 1.5 in coverslips.

### Image sequence collection

Fluorescence movies were collected on a BX51W1 Olympus microscope with Olympus UPlanSApo 60×/1.20 water-immersion objective using an ORCAFlash 2.8 CMOS camera with 5 s integration time. For all samples, at least 60 in-focus frames were collected per view, which varies from sample to sample due to lensing effects on the focus level of individual frames. Sample drift was corrected using the open source ImageJ plugin *Align slices in stack* [35]. Background was subtracted by performing a whole image subtraction of the final frame, which removes any features that remain throughout the entire movie, thus, constituting background, nonspecific binding, or autofluorescent dust particulates.

### Manual SM counting

SM blinking behavior was observed and counted first manually by a trained expert as described in [23] prior to modeling. A grid of 36 μm^2^ squares was overlaid on the image sequence in ImageJ. Pixel regions representing a prospective SM presenting blinking behavior were tagged and summed over each grid. Areal counts were averaged across 12 grids per well and 3 wells per patient and plotted against a standard concentration curve generated by similar analysis of the spiked urine samples.

## Model development

### Model selection

Manual SM counting from the aligned, background-subtracted multi-frame fluorescence micrographs is a time- and resource-intensive sampling method from within the full population of fluorescent molecules in each well. There is a potential to lose valuable SM information carried in grids not sampled, especially at lower concentrations when the presence of only a few molecules per grid may lead to a false negative. Reducing this effort and incorporating full image analysis are of paramount importance to classify SM behavior, improve limits of detection, and increase statistical confidence in derived analyte concentration.

The current experimental dataset presented unique challenges that precluded the use of existing models to identify and quantify SM data. First, the dataset was relatively small in volume. Typical datasets for analysis by machine learning techniques comprise hundreds if not thousands of labeled training examples. Due to the complexity of manual sensor fabrication and FLISA data collection, only a handful of experiments could be performed at any one concentration of lipoarabinomannan (LAM) at one time. Second, the experimental conditions used to generate the data necessarily result in a complex, non-uniform dataset. There was a high degree of variability in both the average SM signal and average background intensities whether considered between frames of one multi-frame fluorescence micrograph or between sets of micrographs, due in part to the variable plasmonic-enhanced electric field above the surface, degree of dye conjugation to antibodies, orientation of molecules and dye-labeled antibodies on the surface, the Gaussian profile of the excitation light, and sample drift.

**Fig 1:**
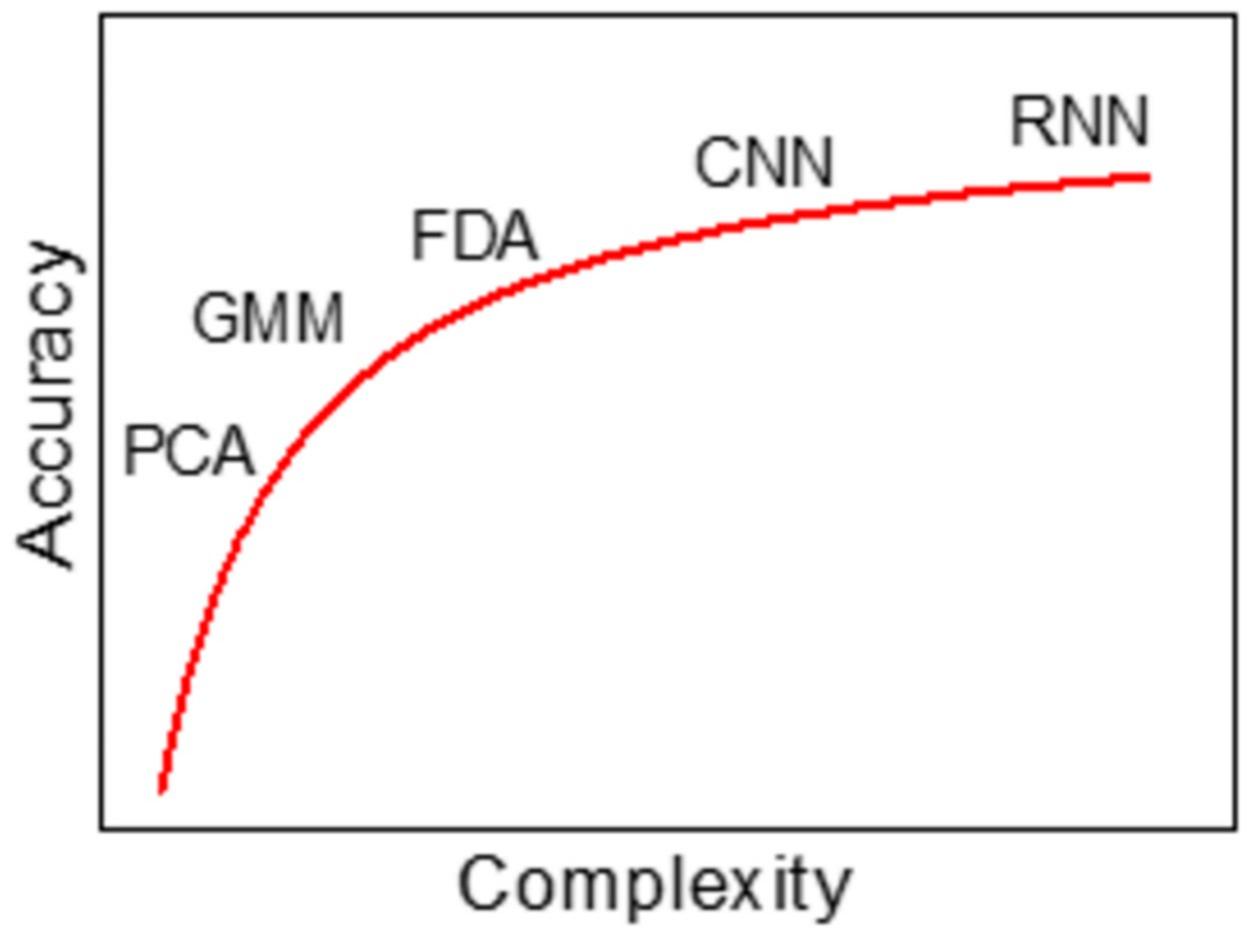
Impact of model complexity on accuracy at convergence.

Any automatic system capable of rapid distinguishing between background and SMs with confidence should have sufficient complexity that SMs are identified correctly yet not so much that computer power and time are sacrificed as to render the method inefficient compared to manual counting. Since manual counting can take up to an hour or more just to sample a few grids from a single multi-frame micrograph, we applied an upper limit time constraint to the overall model design of less than one hour. More rigorous machine learning methods such as GMM (Gaussian Mixture Model), CNN (Convolution Neural Network), RNN (Recurrent Neural Network) and FDA (Functional Data Analysis) may provide greater certainty, but would suffer from the paucity and variability of training data combined with the increase in computation, complexity, and use time per sample (Fig 1).

Meanwhile, simpler methods such as Principal Component Analysis (PCA) are rapid, but lack the sensitivity needed to isolate SMs from a complex background that varies in intensity. Ultimately, we chose to consider a semiautomatic and automatic version of a GMM, the two versions differentiated by whether thresholding prior to classification is performed manually by a user (semiautomatic) or by some automated method such as erosion and dilation (automatic). A semiautomatic model may result in higher model accuracy due to user supervision while saving time and increasing dataset size and confidence compared to manual counting. Meanwhile, an automatic model would generate results faster than any other proposed methods at the expense of requiring increased knowledge of the input dataset and lower sensitivity compared with a semiautomatic model. The decision between the different models to balance this trade-off would be based on our need regarding time and accuracy.

### Analysis of SM behavior

**Fig 2:**
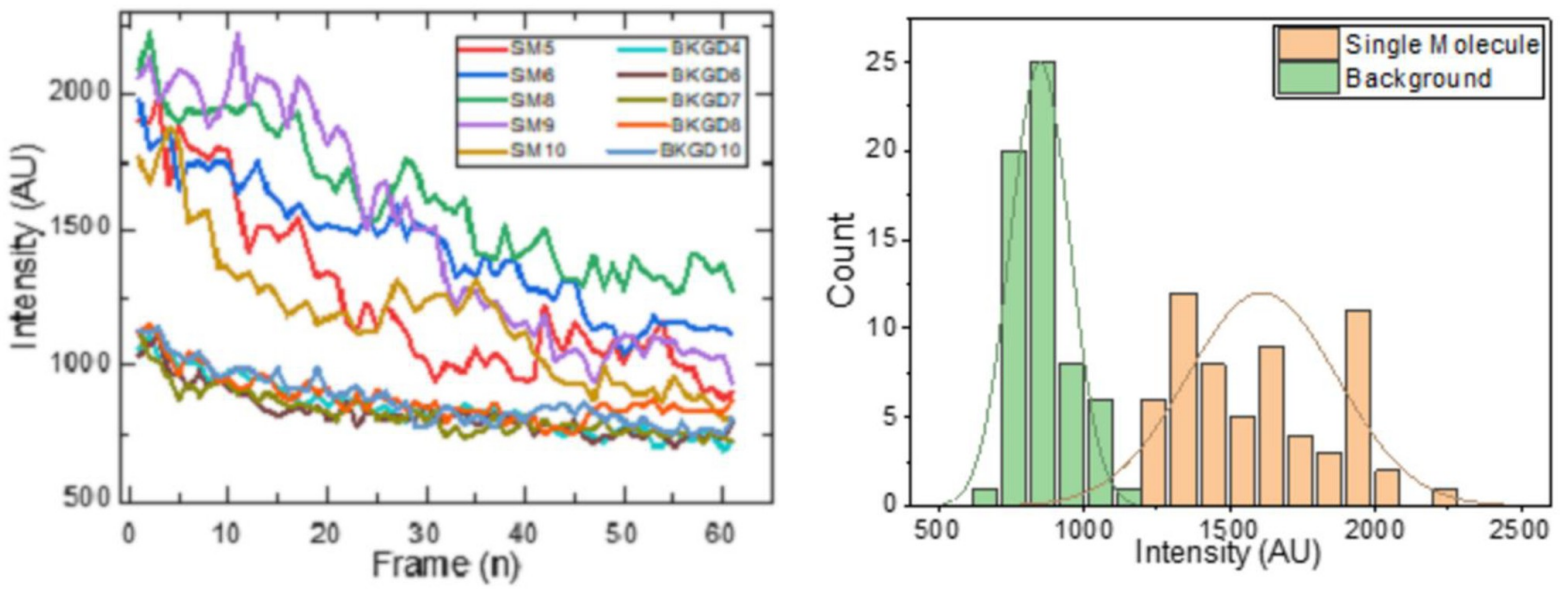
a) Sample multi-frame traces showing behavior of SMs and background regions with no SM and b) Histogram of multi-frame intensity of SM data from LAM dataset.

The multi-frame fluorescent micrographs to be fed into this automatic system comprised sets of sixty 12-bit images with 1920 × 1440 pixels (2,764,800 total pixels). Based on manual analysis in ImageJ, areal counting of SMs per square micron correlated well with the concentration of LAM in the original bulk fluid sample. Visual inspection of a single frame showed SM fluorescence as diffraction limited circular spots from 5–10 pixels in diameter with roughly Gaussian intensity profile. The behavior of SMs and background in this sample with respect to background is demonstrated in Fig 2a. Both SM and regions corresponding to background have intensity that decays with time, a result of the plasmonic enhancement and scattering from the silver surface bleeding into background regions. However, there was still a variable, but observable difference in the intensity of SMs and background. The high variability of SMs resulted from blinking convoluted with the monotonic exponential decay with time. By visual inspection of the image histogram for well-defined SM and background regions, we found that there were two distinct intensity profiles that could roughly be described by Gaussian components (Fig 2b).

Through the analysis of SM behavior, we determined that the main goal of the ML model should be identifying SMs up to the standard set by manual review. In general, a pixel was considered a part of a SM if the pixel had a higher intensity than the identified background threshold and exhibited a blinking pattern across its frames. The ML model was designed to identify which pixels were a part of SMs (i.e., elevated intensity, blinking), use a counting algorithm to combine adjacent pixels, and count SMs based on those pixels. Eventually, the ML model yielded a total number of SMs contained in the processed image and provided a count of molecules per square micrometer.

### Model 1: Semiautomatic SM counting method

**Fig 3:**
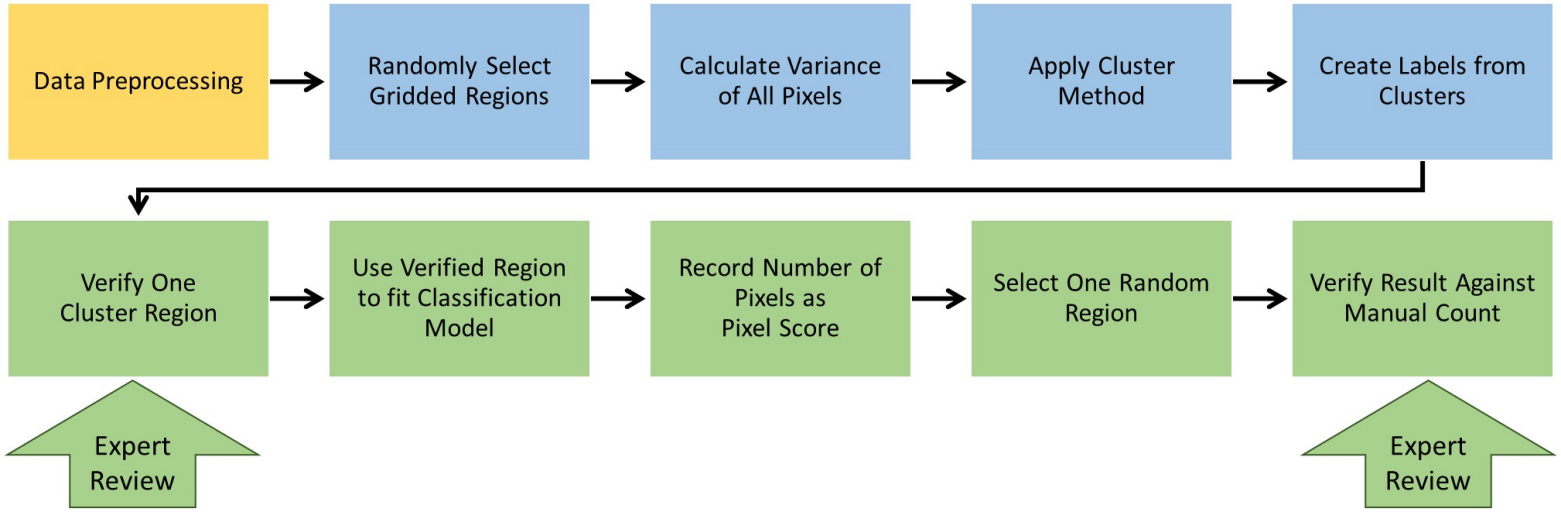
Flowchart of semiautomatic model of SM counting. From text, steps are label (orange), classify (blue), and review (green).

The semiautomatic SM counting method (Fig 3) is broken down into three steps: label, classify, and review.

#### Label

The input data, a multi-frame fluorescence micrograph, was segmented into 100 image segments of 172 × 124 × N, where N was the number of frames in the micrograph. Using a Gaussian Mixture Model (GMM) clustering algorithm, a label was assigned to every pixel in 40 randomly selected image segments. Next, four image segments were randomly selected for expert comparison with the original images. If the labeled pixels in the selected image segments were considered accurately matched with the expert-identified SMs, we proceeded to the classification step. Otherwise, the expert could choose either to change the cluster method or tune model parameters to improve the sensitivity and selectivity of the model.

#### Classify

Labeled pixels in the 40 test image segments were used to train a classification model called gradient boosting. Note that the parameters used in this step include the pixel spatial coordinates and grayscale intensity value from pixels in frames 15–40 of each multi-frame fluorescence micrograph. The remaining 60 image segments were then fed into the trained model to classify them as containing SMs or not.

#### Review

Six of the 60 image segments above were randomly chosen to present to the expert for final review. If the results did not match with expert user classification of ‘SM’ and ‘not SM’, new classification methods were chosen to retrain the model until results were deemed acceptable to the experts.

Note, we have also tried K-means for clustering and random forest for classification for our Model 1. However, GMM (for the clustering step) and gradient boosting (for the classification step) gave the best performance.

### Model 2: Automatic SM counting method

**Fig 4:**
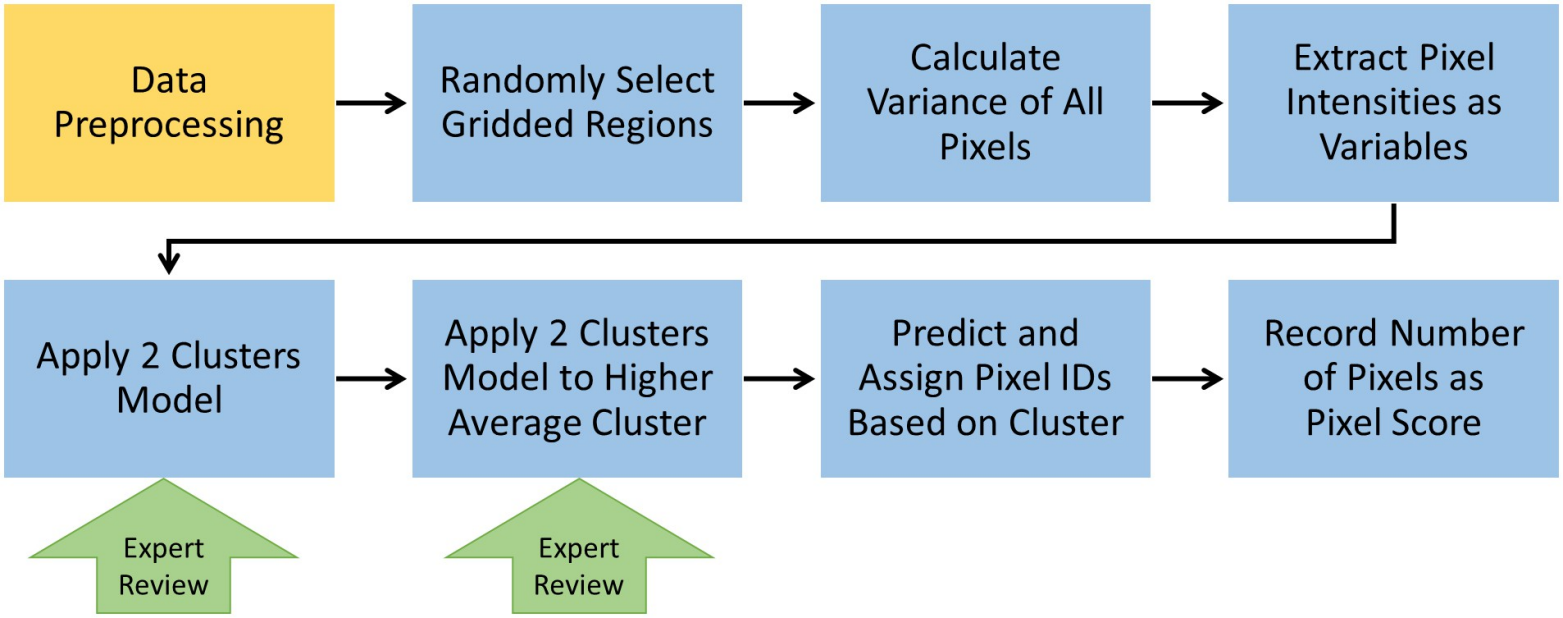
Flowchart of automatic model of SM counting. From text, steps are pre-processing (orange) and modeling (blue), and expert review for quality control only (green).

The automatic SM counting method (Fig 4) consists of two steps: pre-processing and modeling.

#### Preprocessing

The expert first checked the data and provided recommendations for the proper preprocessing method such as a threshold cut, erosion and dilation, or more sophisticated methods. Next, the variance of different pixel intensities across all frames was calculated and the pixel intensities of frames 20, 25, and 30 were recorded to give more dimension to each data point so that the cluster model could detect the connections between data points more clearly. Preprocessing could also reduce the number of pixels fed into the cluster model to save more time.

#### Modeling

During our study, it became apparent that a third population could be isolated as distinct from ‘molecule’ with higher, variably blinking pixel intensity and ‘not molecule’ with lower/background pixel intensity: the high pixel intensity, less variable or non-blinking fluorescence of nonspecific binding of fluorescently labeled antibodies and of proteins containing aromatic amino acid residues. This means there must exist at least three clusters in the data rather than the assumed two, namely, background, SMs, and nonspecific binding. Having chosen to use K-means clustering for Model 2, there were two ways to implement clustering on this data, either apply a three-cluster model directly or apply a two-cluster model twice–first to isolate all molecules from background and the second to isolate nonspecific binding from SMs. In general, we found that the 2 × 2-step cluster model resulted in higher accuracy. Manual inspection was used to review the accuracy of the predicted count in a manner similar to the semiautomatic model, which could be removed in the field deployment of this model.

### Clustering methods

#### Gaussian mixture

A GMM was implemented to analyze the whole multi-frame fluorescence micrograph. GMM presents data by assuming there is a finite number of ‘components’ that follow Gaussian distribution described by a component mean and variance. The Expectation-Maximization (EM) Algorithm [36] evaluates the mean and variance of each component and uses their average mean and variance to generate clusters and create a cluster map. Visual inspection above identified two distinct components, namely, ‘SMs’ and ‘*not* SMs’ or background.

Algorithm:

1. Start with random initial estimates of distributions, each with their associated mean ***μ***_***k***_, variance ***σ***_***k***_ and mixture weight ***α***_***k*,**_ which represent the probability that randomly selected data point *x*_*i*_ was generated by cluster *k*.
2. E-steps: use current mean ***μ***_***k***_, variance ***σ***_***k***_, and mixture weight ***α***_***k***_ to compute membership weight ***W***_***ik***_ of data point *i* in cluster *k*.
3. M-steps: use membership weight ***W***_***ik***_ to recalculate new value for mean ***μ***_***k***_, variance ***σ***_***k***_, and mixture weight ***α***_***k***_ of cluster *k*.
4. Repeat E-steps and M-steps until the change in new value is below required threshold.

After the EM Algorithm stabilizes, the fitted GMM model is used to calculate the log of membership weights and the log of mixture weights for each data point *i* in cluster *k*, log(***W***_***ik***_) + log(***α***_***k***_). For each data point, the cluster *k*_*max*_ that maximizes *log(****W***_***ik***_*) + log(****α***_***k***_*)* will be the cluster to which that particular data point is finally assigned.

#### K-means

The K-means [37] model was designed to identify different functional patterns among variables across time. Let *X*_*n*_ be the dataset to be analyzed, *V*_*c*_ be the set of cluster centers in *X*_*n*_ in *m* dimensional space, *n* the total number of objects, and *c* the number of clusters. For the multi-frame fluorescence micrographs, *X*_*n*_ is a vector of pixel values, *V*_*c*_ is randomly assigned from *X*_*n*_, *m* is the number of frames, *n* is the image size, and *c* is 3. The goal of the algorithm is to minimize any types of distance between *X*_*n*_ and *V*_*c*_, namely, the Euclidean distance between the two values.

Algorithm:

1. Functional Data K-means Algorithm: Centroids of 2 clusters *V* are chosen from *X*
2. Calculate between *V* and *X*
3. Reassign *X* to its closest *V*
4. Repeat and update *V* via:
5. Repeat steps 2–4 until no data points are assigned to new clusters.

### Classification method

#### Gradient boosting

Gradient boosting is an algorithm designed to optimize boosting type classification tree models [36, 38]. The grayscale values of each pixel in each frame will serve as variables to be processed. The goal of the model is to have a minimized mean squared error between predicted and real values.

Algorithm:

Input: Training set {(*X*, *Y*)}, and a differentiable loss function *L(y*, *F(x))*, with iterations number *M*.

Steps:

1. Initialize the model with constant values:
$$F_{0}\left(x\right)=argmin_{ϒ}\sum_{\text{n}=1}^{\text{n}}\text{L}\left({\text{y}}_{\text{i}},\gamma \right)$$
2. For *m = 1* to *M*:
  - Compute the pseudo-residuals:
$$r_{im}=-{\left[\frac{\partial L\left(y_{i},\;F\left(x_{i}\right)\right)}{\partial \text{F}\left({\text{x}}_{\text{i}}\right)}\right]}_{F\left(x\right)=F_{m-1}\left(x\right)}$$
  - Fit a base learner *h*_*m*_*(x)* to pseudo-residuals:
  - Compute multiplier γ_m_ by solving following function:
$$\gamma_{m}=argmin_{ϒ}\sum_{\text{n}=1}^{\text{n}}\text{L}\left({\text{y}}_{\text{i}},\;F_{m-1}\left(x_{i}\right)\;+\;\gamma h_{m}\left(x_{i}\right)\right)$$
  - Update the model:
$$F_{m}\left(x\right)=F_{m-1}\left(x\right)\;+\;\gamma_{m}h_{m}\left(x\right)$$
3. Output *F*_*m*_*(x)*

#### Random forest

Random forest is an improvement on the bootstrap aggregating tree model [39]. It gains accuracy as the tree grows deeper without suffering from overfitting [40]. Like gradient boosting, the grayscale values of each pixel of each frame are variables so that the model can minimize mean squared error.

Algorithm:

Input: Training set *{X}* with its response *{Y}*.

Steps:

1. Bagging them B times (selects a random sample with replacement). New sample is called *X*_*b*_ and *Y*_*b*_.
2. For *b = 1*,*…*,*B*: train a classification tree *f*_*b*_ on *X*_*b*_ and *Y*_*b*_.

Output:

Model prediction is based on average of all individual tree *f*_*b*_

### Counting method

A follow-up counting algorithm [41, 42] performs after training is done:

1. Consider all pixel values are *p*_*i*_
2. For *i = 1* to *N*:
  If *p*_*i-1*_ *= p*_*i+1*_ *= p*_*i-172*_ *= p*_*i+172*_ *= p*_*i*_ *≠ 0* (0 is always defined as background) create a cluster and assign a label to it.
3. Repeat
4. Count the number of labeled points, which corresponds to the number of SMs.

Note: 172 is the width of input image. *N* in our case is the number of pixels in our image. If i-172 is less than 0 or *i+172* is larger than 21500, it will be ignored.

Since the threshold or cutoff parameter plays such a pivotal role in isolating SMs and classifying them properly, this counting algorithm was used in both the semiautomatic and automatic models.

## Results

### Implementing FLISA analysis by semiautomatic and automatic models

**Fig 5:**
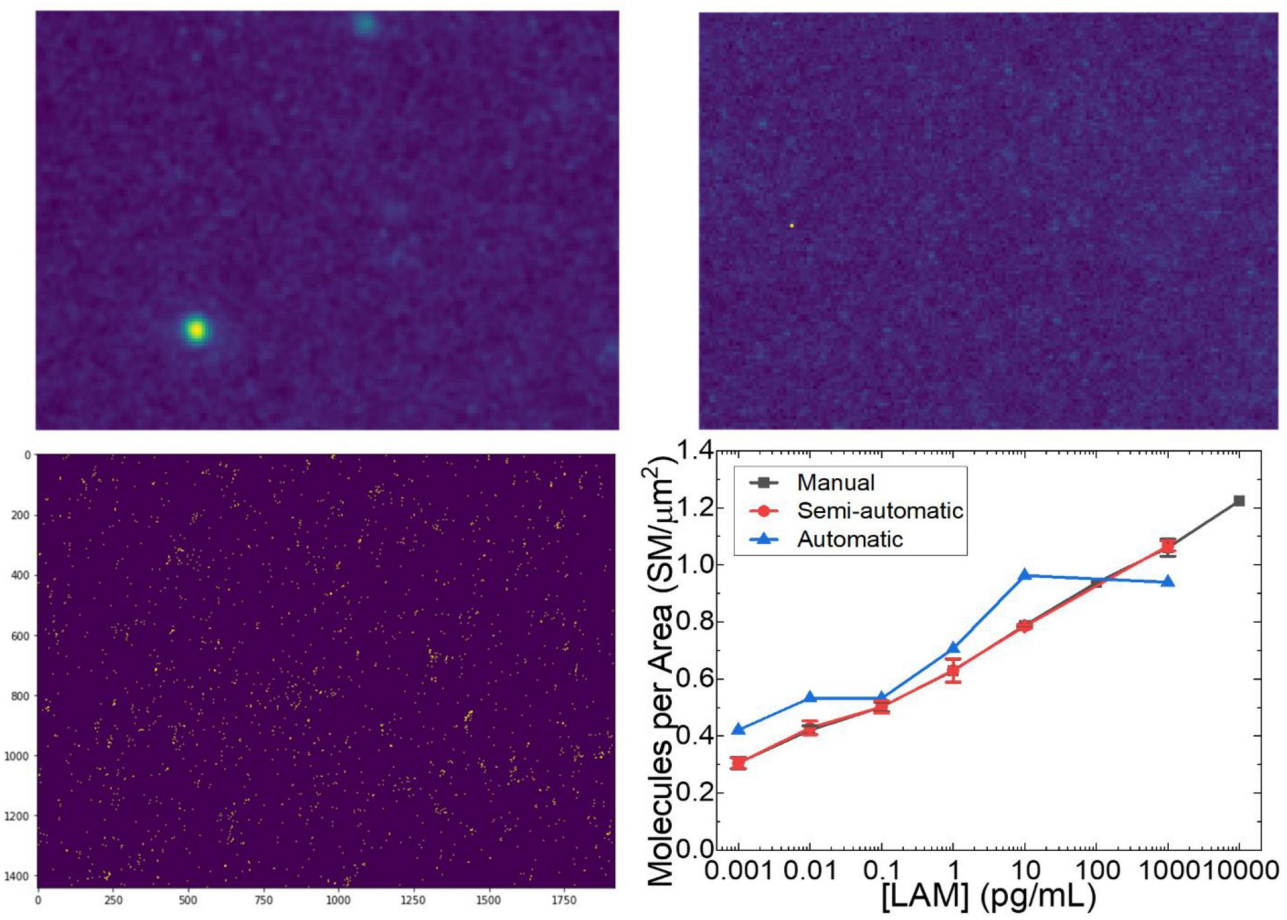
a) Sample 172 × 124 × 1 image segment with a few potential SMs surrounded by pixelated background. b) Sample image segment with no apparent potential SMs suggesting only background. c) Example single frame fluorescence micrograph after application of manual thresholding during semiautomatic GMM Model 1. d) Result of semiautomatic GMM Model 1 and automatic Model 2 compared to the manual count.

Analysis of FLISA data by manual counting typically takes around 1–2 hours per micrograph or 12–24 hours per dataset depending on a variety of personnel and experimental factors such as expertise, signal-to-noise ratio, image clarity due to microscope focus, and number of molecules present. Edge cases such as extremely low or high concentrations of SMs present challenges to manual counting that the user must spend significant time to overcome. Fig 5 contains sample 172 × 124 × 1 pixel image segments representing regions with (Fig 5a) and without (Fig 5b) likely candidate SMs. By manual counting, the expert user or technician would be required to tag the potential SMs in one frame and then check multiple adjacent frames for the presence of apparent SM blinking. Even after streamlining this process, detection of SMs in a few sampled grids per image set could only be reduced to several days to complete analysis of a whole dataset of triplicate physical samples per concentration and multiple concentrations of LAM.

Meanwhile, implementing semiautomatic model 1, including ‘expert’ thresholding, took only ~1.5 hours to complete the same dataset on a regular laptop. Similarly, automatic model 2 typically completes its runtime around 45 minutes, confirming that similar accuracy can be achieved at a fraction of the time and resource allocation. To be more specific, our GMM and gradient boosting method took 15 minutes to process 1 sample. Meanwhile, FDA took 2 hours to process 1 sample, CNN took 2.5 hours, and RNN took 3 hours as shown in S1 Table. Fig 5c shows a representative single frame fluorescence micrograph demonstrating application of the manual thresholding algorithm. From this converted binary image, molecules were counted considering each grouping of adjacent ‘on’ pixels as one molecule. For automatic model 2, consistent results were produced by implementing a percentile cut thresholding image pre-processing step of 98.5 percentile for 1 fg/mL and decreasing by 0.25–0.375 per step. Applying this thresholding to more frames within each image set enabled counting of molecules that may be in the ‘off’ or dark state in the first frame and blink ‘on’ or fluoresce in later frames, increasing the number of molecules identified per sample and achieving more accurate results.

Fig 5d compares the results of manual counting of SMs with the results of semiautomatic model 1 with GMM (for clustering) and Gradient Boosting (for classification) combination and automatic model 2 using K-means algorithm. Because the expert thresholding step is equivalent to the mental thresholding performed during manual visual inspection, the result of the semiautomatic model was found to be a very close approximate match to the manual count. A similar trend in the data was found using the automatic model, although the number of molecules counted per concentration was uniformly higher than by both manual count and semiautomatic model 1 except for 1000 pg/mL (or 1 ng/mL). The increase in counted molecules across lower concentrations is thought to be due to edge cases near the threshold cutoff where automatic thresholding might produce nearly adjacent ‘molecules’ comprising one or two pixels and count them as separate molecules instead of one molecule. Meanwhile, further examination of the automatic model revealed that pixels that may represent SMs were cut by the automatic thresholding step prior to the counting algorithm as a result of the increasing background intensity at concentrations near the transition from SM blinking to bulk fluorescence between 100–1000 pg/mL.

### Calibration curve analysis

Transition from bulk fluorescence emission to SM emission behavior was observed between 1 ng/mL and 100 pg/mL LAM concentration, as evidenced by the evolution of blinking behavior across those decades of concentration. Overall, a significant difference (95% confidence interval) was obtained between samples with 2-orders of magnitude difference in LAM concentration (e.g., 1 fg/mL and 100 fg/mL), but not with 1-order of magnitude difference in LAM concentration. As discussed in [23], a log-linear function was found to best fit the data collected across the two days of sensor experiments and equations were generated to provide a standard curve for each group with adjusted R^2^ = 0.994 and 0.9844, respectively. The same calibration curve generation process was performed using the semiautomatic and automatic analyzed data with resulting R^2^ = 0.991 and 0.776, respectively (Table 1). The R^2^ of the automatic calibration curve function is lower due to the presence of an errant datapoint at 1000 pg/mL or 1 ng/mL. As mentioned above, this concentration is around the transition point from SM to bulk fluorescence, so SMs begin to appear to coalesce within the fluorescence image, leading to an undercount in the current iteration of the automatic model. The curve coefficients for each group control set are in good agreement despite the tests being performed roughly 1 week apart and on different grating substrates, an important validation of the consistency of the grating sensor surface and the reproducibility of the SM counting method to determine LAM concentration. These equations were later used to translate the number of molecules per grid area of the patient samples into corresponding LAM concentrations per milliliter initial volume.

**Table 1:** Fit parameters for LAM concentration standard curves.

Fitted Equation: Y = A–B* ln(X+C)				
Molecules per μm^2^ (Y) vs [LAM] (X)				
	A	B	C	R^2^
**Semiautomatic**	0.651 ± 0.013	-0.059 ± 0.003	0.002 ± 0.002	0.991
**Automatic**	0.715 ± 0.050	-0.042 ± 0.013	5E-6 ± 0.004	0.776

### Patient sample analysis

**Fig 6:**
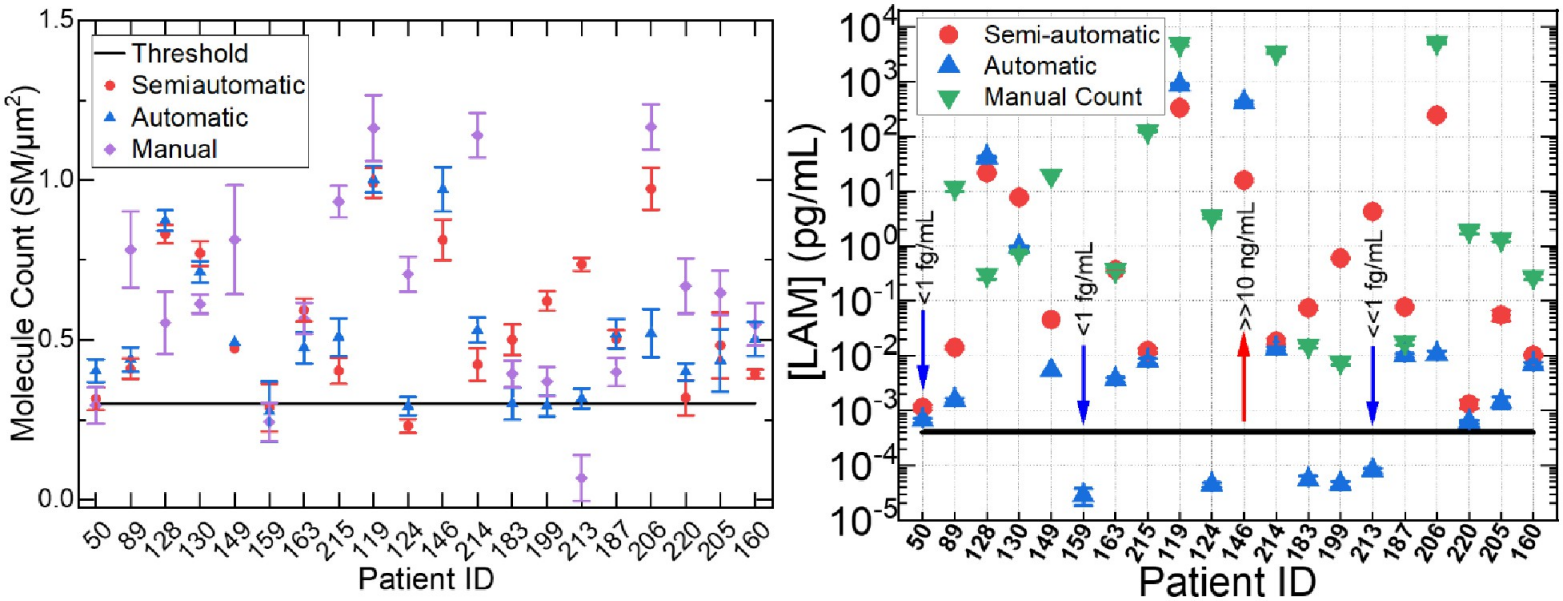
Comparison of results of semiautomatic and automatic models with manual count: a) molecular count per square micrometer and b) converted LAM concentration in picograms per milliliter.

Test data were acquired from 20 clinical patient sample urines, eighteen of which were confirmed ‘ground truth’ positive with TB by manual count of the FLISA data and by collaborators in a previous work [23]. These data are summarized alongside the results of the semiautomatic and automatic model results in Table 2. The areal counts and converted calibrated molecular LAM concentration are also represented graphically in Fig 6a and 6b, respectively. The dynamic range is considered to be from the bulk fluorescence emission transition at 1000 pg/mL (or 1 ng/mL) to the lower threshold limit of detection, 0.001 pg/ml level (or 1 fg/mL), below which we consider any fluorescence to be nonspecific binding. Any SM/μm^2^ above that threshold is considered to represent a TB-positive sample. According to the ground truth diagnosis by both Gene XPert and cell plating, patients 50 and 159 were declared negative for TB. This is evidenced by the low molecular count and corresponding converted LAM concentration for these patients. The semiautomatic model using the combination of GMM and gradient boosting registered both patient 50 and 159 as at or below the threshold level while the automatic model using GMM registered patient 159 below threshold, but registered patient 50 as having above threshold level of LAM molecules. GMM and random forest yielded similar results at significantly slower run-time. The incorrect assignment of patient 50 by the automatic model was due to the preprocessing step and proteinuria in the patient 50 sample, which resulted in uncharacteristically high nonspecific binding. Both the semiautomatic and automatic models incorrectly identified patient 124 as being TB-negative, but correctly identified patient 213 as at or above the threshold for TB where the manual count did not. Additionally, patient 146 only had one usable multi-frame image as a result of a microscopy error, so there is no coefficient of variation for that result. The results of semiautomatic model 1 provided 88.89% clinical sensitivity, while the automatic model resulted in 77.78% clinical sensitivity. In general, the semiautomatic model 1 tends to have the lowest coefficient of variance of all three methods. If precision is a prior consideration, the automatic model produced the worst result.

**Table 2:** Comparison of SM counting by semiautomatic model, automatic model, and manual count with ground truth diagnosis.

Patient ID	Semiautomatic Count (SM/μm^2^ ± SD)	Coeff Var	TB Status (Semi)	Automatic Count (SM/μm^2^ ± SD)	Coeff Var	TB Status (Auto)	Manual Count (SM/μm^2^ ± SD) [23]	Coeff Var	TB Status (Manual) [23]	Ground Truth Diagnosis [23]
50	0.316 ± 0.036	0.114	-	0.404 ± 0.036	0.089	+*	0.296 ± 0.057	0.193	-	-
89	0.410 ± 0.031	0.076	+	0.440 ± 0.038	0.086	+	0.783 ± 0.120	0.153	+	+
128	0.831 ± 0.029	0.035	+	0.874 ± 0.032	0.036	+	0.554 ± 0.097	0.176	+	+
130	0.771 ± 0.039	0.050	+	0.713 ± 0.033	0.046	+	0.613 ± 0.030	0.049	+	+
149	0.473 ± -	--	+	0.493 ± -	--	+	0.814 ± 0.171	0.210	+	+
159	0.289 ± 0.074	0.255	-	0.276 ± 0.095	0.343	-	0.244 ± 0.059	0.243	-	-
163	0.593 ± 0.036	0.060	+	0.476 ± 0.049	0.104	+	0.567 ± 0.049	0.087	+	+
215	0.404 ± 0.401	0.099	+	0.509 ± 0.059	0.116	+	0.933 ± 0.049	0.053	+	+
119	0.991 ± 0.047	0.047	+	1.003 ± 0.042	0.041	+	1.163 ± 0.103	0.089	+	+
124	0.231 ± 0.022	0.094	-*	0.293 ± 0.029	0.099	-*	0.706 ± 0.055	0.078	+	+
146	0.813 ± 0.063	0.077	+	0.971 ± 0.070	0.072	+	>> 10 ng/mL	--	+	+
214	0.424 ± 0.051	0.120	+	0.531 ± 0.040	0.075	+	1.141 ± 0.069	0.061	+	+
183	0.501 ± 0.048	0.095	+	0.301 ± 0.050	0.165	-*	0.394 ± 0.041	0.104	+	+
199	0.622 ± 0.031	0.050	+	0.294 ± 0.033	0.113	-*	0.370 ± 0.045	0.122	+	+
213	0.736 ± 0.021	0.028	+	0.317 ± 0.031	0.097	+	0.068 ± 0.072	1.059	-*	+
187	0.503 ± 0.028	0.056	+	0.519 ± 0.046	0.089	+	0.400 ± 0.044	0.111	+	+
206	0.973 ± 0.066	0.068	+	0.522 ± 0.075	0.144	+	1.167 ± 0.071	0.061	+	+
220	0.320 ± 0.055	0.173	+	0.401 ± 0.026	0.066	+	0.669 ± 0.086	0.129	+	+
205	0.483 ± 0.103	0.213	+	0.437 ± 0.098	0.225	+	0.648 ± 0.069	0.106	+	+
160	0.394 ± 0.014	0.035	+	0.503 ± 0.052	0.103	+	0.549 ± 0.066	0.120	+	+

## Discussion

In summary, we have demonstrated a SM fluorescence imaging assay for ultrasensitive detection of disease biomarkers from noninvasive body fluids that takes advantage of machine learning techniques to improve accuracy and reduce the necessity of interference by and workload of expert users. Clustering methods such as GMM were used to identify and detect SMs from the fluorescence images, enabling the detection of LAM from a set of unprocessed images of clinical patient urine samples. Both a semiautomatic and automatic version of the model were implemented, and both identified multiple distributions of molecules that were distinct in features, such as fluorescence intensity, blinking frequency, and photobleaching rate. The results of these two methods were compared to the previously confirmed manual count by experts. The supervised learning algorithm (i.e., the semiautomatic model with GMM and gradient boosting) outperformed the unsupervised learning algorithm (i.e., the automatic model with K-means), demonstrating the continued need for expert intervention to overcome the complexity of the fluorescence image samples with such a small size training dataset. This analysis and positive determination of TB infection could be strengthened by performing the gold standard cell culture on all samples rather than just to confirm negative TB status. Collecting a statistically larger dataset in this manner is the subject of future study.

The primary benefits of semiautomatic model 1 with respect to the manual count derive from the integration of the accuracy of the human eye with the speed of modern computing. Even accounting for the remaining expert intervention time and post-count quality control review, the time-to-detection was reduced by more than 85% because of instantaneous application of the thresholding step through the user interface. Furthermore, while expert analysis was performed over a small fraction of each image set (roughly 1/60–1/50 of each image), sampling was increased to integrate the whole micrograph using the ML model. This analytical process reduced potential human bias because the entire image set was used rather than selecting a portion of the data. Meanwhile, automatic model 2 provided a result with overall faster speed and allowed the machine to handle data independently of any expert user, allowing any person to rapidly identify SMs from the fluorescence micrographs with minimal input or training. However, the automatic model result does not match as closely with the manual count result as does the semiautomatic model 1, the mismatch of which may potentially cause an unacceptable error in diagnosis. The greatest challenge with implementing the automatic model remains accurate thresholding from such a complex and dynamic dataset, percentile cutting serving to approximate the discernment of an expert user with mixed results at edge case concentrations of LAM.

We envisage that the automatic model may outperform the semiautomatic model when there are sufficient labeled training data for the automatic ML algorithm. In this work, the training data was limited to only triplicates of each distinct datapoint due to the research scale at which the FLISA grating was fabricated and assayed. In future, this assay and associated models could be expanded on a number of fronts. First, the SM fluorescence data could be derived from other imaging sources capable of SM detection, such as Total Internal Reflection Fluorescence (TIRF) microscopy, Photoactivated Localization Microscopy (PALM), and others [43, 44]. Next, the FLISA platform may be modified to detect a number of disease state biomarkers, including the emerging COVID-19, which has been detected from noninvasive sources such as feces, but has so far been difficult to isolate from urine by culture or real-time polymerase chain reaction (RT-PCR) testing [45, 46]. Moreover, the platform and algorithms could be used to detect the presence and quantity of human antibody to indicate infection and antibody isolates could be screened through SM imaging for interaction with disease biomarkers of interest for titer determination, viral serotyping, and vaccine production. Expanding further, the ML algorithms could be applied to other SM imaging applications, with presumably similar improvements in computational time and analytical accuracy.

## Supporting information

S1 Table The running time and clinical sensitivity of different models for the analysis. (DOCX)
