# Supplementary material for: Applications of machine learning tools for ultra-sensitive detection of lipoarabinomannan with plasmonic grating biosensors in clinical samples of tuberculosis
Source: PLoS One. 2022 Oct 25;17(10):e0275658. doi: 10.1371/journal.pone.0275658 (PMC9595565; doi:10.1371/journal.pone.0275658)
Supplement: S1 Table — (DOCX) [file pone.0275658.s001.docx]

**Supporting Information**

**S1 Table.** The running time and clinical sensitivity of different models for the analysis

| Model | Run time (per sample) | clinical sensitivity |
| --- | --- | --- |
| GMM and Gradient boosting semiautomatic combination | 15 mins | 88.89% |
| K means automatic method | 7-8 mins | 77.78% |
| K means and FDA semiautomatic combination | ~2 hours | 83.33% |
| K means and CNN semiautomatic combination | ~2.5 hours | 88.89% |
| K means and RNN semiautomatic combination | ~3 hours | 88.89% |

**Note.** All models are calculated under a cluster with 4 1080ti GPU.
